# SPEAR Trial: Smartphone Pediatric ElectrocARdiogram Trial

**DOI:** 10.1371/journal.pone.0136256

**Published:** 2015-08-21

**Authors:** Hoang H. Nguyen, George F. Van Hare, Michael Rudokas, Tammy Bowman, Jennifer N. A. Silva

**Affiliations:** Washington University in St. Louis School of Medicine, Department of Pediatrics/Division of Cardiology, St. Louis, Missouri, United States of America; The Ohio State University, UNITED STATES

## Abstract

**Objectives:**

Smartphone-enabled ECG devices have the potential to improve patient care by enabling remote ECG assessment of patients with potential and diagnosed arrhythmias. This prospective study aimed to assess the usefulness of pediatric ECG tracings generated by the AliveCor device (Oklahoma City, OK) and to assess user satisfaction.

**Study Design:**

Enrolled pediatric patients with documented paroxysmal arrhythmia used the AliveCor device over a yearlong study period. Pediatric electrophysiologists reviewed all transmitted ECG tracings. Patient completed surveys were analyzed to assess user satisfaction.

**Results:**

35 patients were enrolled with the following diagnoses: supraventricular tachycardia (SVT, 57%), atrial fibrillation (AF, 11%), ectopic atrial tachycardia (EAT, 6%), atrial tachycardia (AT, 3%), and ventricular tachycardia (VT, 23%). A total of 238 tracings were received from 20 patients, 96% of which were of diagnostic quality for sinus rhythm, sinus tachycardia, SVT, and AF. 126 patient satisfaction surveys (64% from parents) were completed. 98% of the survey responses indicated that it was easy to obtain tracings, 93% found it easy to transmit the tracings, 98% showed added comfort in managing arrhythmia by having the device, and 93% showed interest in continued use of the device after the study period ended.

**Conclusions:**

Smartphone-enabled ECG devices can generate tracings of diagnostic quality in children. User satisfaction was extremely positive. Use of the device to manage certain patients with AF and SVT showcases the future role of remote ECGs in the successful outpatient management of arrhythmias in children by potentially reducing Emergency Department visits and healthcare costs.

## Introduction

Cardiac arrhythmias are a common cause of morbidity in both the adult and pediatric populations. The Task Force on Children and Youth estimate that annually 30,000 children either develop a cardiac arrhythmia or are born with a conduction abnormality [1]. An additional 32,000 children each year are born with a cardiac malformation, many of who (~25–30%) may also have conduction anomalies [1]. Since pediatric arrhythmias are predominantly paroxysmal in nature, their detection relies primarily on the arrhythmias being captured on happenstance electrocardiograms often making diagnosis an inconvenient and expensive process. Most often pediatric cardiologists rely on 24-hour ambulatory monitors, external event monitors, or implantable loop recorders (ILRs) to evaluate children with cardiac arrhythmias. However, these devices only offer a finite period of use sometimes necessitating repeated uses to record transient arrhythmias. They can also be expensive (up to $4000) and burdensome (surgical intervention for placement of an ILR, continuous wear of an external device) to patients [2]. Furthermore, some of these devices do not provide real time access to transmitted ECG tracings leading to potential delay in diagnosis and potential unattended life threatening events. This in turn can cause anxiety and distress for patients and parents. A more effective and cost efficient monitoring technology would need to be easily incorporated into users’ daily life, to be available on demand and promote the direct connection between users and their physicians.

One such novel technology is the real time smartphone-enabled ECG that combines minimal external hardware (plate containing electrodes) with a smartphone application and Wi-Fi connectivity or data enabled smartphone plan that allow patients to capture and transmit single-lead (lead I or lead II) ECG tracings directly to their physicians. An ECG tracing (lead I) is obtained by placing a finger of each hand on each of the electrode or by placing the electrodes directly on the chest. Alternatively, one can put one electrode on the left knee and a finger of the right hand on the other electrode (lead II). The electrodes detect electrical signal on the surface of the skin and convert it to an ultrasonic signal that is in turn picked up by the smartphone’s microphone. The tracing can be viewed in real time while being recorded. The tracing is stored locally on the smartphone, and it is also automatically transmitted to AliveCor’s secure, encrypted servers over Wi-Fi or data enabled cellular network. The tracing can be emailed directly from the smartphone application to the physician for review. Alternatively the physician can review a patient’s tracing by using the company’s web-based application. This technology has been validated in the screening for atrial fibrillation [3]. Despite its clear potential to improve patient care by enabling remote ECG tracings assessment in patients with potential and diagnosed arrhythmias, there have been no studies to date in the pediatric population. This trial was initiated to: 1) assess the usefulness of pediatric ECG tracings generated by the US Food and Drug Administration (FDA) approved AliveCor device (AliveCor Inc., Oklahoma City, OK), and 2) assess user satisfaction with the device.

## Methods

This prospective study received full approval from the institutional review board of the Washington University in St. Louis School of Medicine. Pediatric patients with the following inclusion criteria were enrolled from 9/27/2013 to 9/27/2014: 1) age ≤ 18 years, 2) documented paroxysmal arrhythmia, 3) owning an iPhone 4/4S/5, and 4) English speaking. Enrolled patients did not receive any direct financial rewards. AliveCor provided the devices used in this study. Written informed consent was obtained from patients or their families. Written assent consents were obtained from children older than 8 years of age. Our institutional review board approved the protocol.

After consent was obtained, patients and parents were instructed on how to use the device. Users were able to download the application and recorded a test strip. During the study period, users obtained ECG tracings at home while the patients were having symptoms, due to parental concerns, or merely for routine surveillance at the recommendation of the research team or at parental discretion. Users were instructed to transmit ECG tracings of concern to the research team via e-mail. To ensure prompt review of the transmitted tracings, users were instructed to notify the research team of a new transmission by contacting the cardiology office during business hours and the cardiology staff on call after hours. Pediatric cardiac electrophysiologists reviewed all tracings within minutes of being notified. All users were contacted by e-mail and/or telephone with the tracing interpretations and further care instructions. Finally, users were asked to complete online surveys regarding their experience with the device. Users were asked to complete a survey within 24 hours of a transmission. In addition they are also asked to complete monthly surveys. Collection of repeated survey data was aimed to track user experience not only related to a specific transmission but also over time since some users might be using the device but not transmitting.

Users were emailed a private link to the survey using surveymonkey.com (Survey Monkey Inc., Palo Alto, CA, USA). The online survey consisted of 12 questions. Initial questions were used to collect demographic and clinical data, identify the individual who transmitted the tracings, and record the preferred placement of the device. Participants were also asked if they had transmitted any tracings within the past 24 hours to determine whether the survey reflected user experience related to a recent transmission. Usage data were evaluated using a 5-point Likert scales with questions about the frequency of recordings and the frequency of transmissions. User experience was also evaluated with 5-point Likert scales with questions to assess the ease of obtaining a tracing, the ease of transmission of a tracing, the level of comfort in managing arrhythmia with the help of the device, and the level of interest in continued use of the device after completion of the study. The study-designed questionnaire was not validated for statistical significance.

In addition to descriptive analysis of the survey responses, data were also analyzed to compare differences between the group of users who transmitted ECG tracings and the group of users who did not transmit. Analysis was performed using Survey Monkey (Survey Monkey Inc., Palo Alto, CA, USA) for the descriptive statistics and SPSS statistical software (version 22.0; IBM Corp., Armonk, NY) for chi-square statistics.

## Results

### Demographic Data

In total, 35 patients were enrolled during this 1-year study. The median follow up time was 8 months (3–12 months). There were almost equal numbers of male and female patients (Table 1) with a median age of 12 years (2 weeks–18 years). Eighteen patients were younger than 12 years, with 9 patients being ≤4 years old. Patients carried the following diagnoses: supraventricular tachycardia (SVT, n = 20, 57%), atrial fibrillation (AF, n = 4, 11%), ectopic atrial tachycardia (EAT, n = 2, 6%), atrial tachycardia (AT, n = 1, 3%), and ventricular tachycardia (VT, n = 8, 23%). At the time of enrolment, 13/35 (36%) patients were not on any antiarrhythmic therapy, 16/35 (46%) were on beta blockers, 2/35 (6%) were on amiodarone, 1/35 (3%) was on flecainide, 1/35 (3%) was on verapamil, 1/35 (3%) was on amlodipine and atenolol, and 1/35 (3%) was on amiodarone and digoxin.

**Table 1:** Patient characteristics.

Patient Characteristics	Number (range or %)
Number of patients	35
Median age (years)	12 (2 weeks-18 years)
Number of patients <12 years	18 (50%)
Number of patients <4 years	9 (25%)
Number of female patients	17 (49%)
Supraventricular tachycardia	20 (57%)
Atrial fibrillation	4 (11%)
Ectopic atrial tachycardia	2 (6%)
Atrial tachycardia	1 (3%)
Ventricular tachycardia	8 (23%)

### Transmission Data

A total of 240 tracings were received from 20 patients. Fifteen patients did not transmit. The maximum number of tracings received from one user during a day was 14. Of the 238 transmitted tracings, 231 (96%) were of diagnostic quality. Nine of the 238 (4%) transmitted tracings were not interpretable because of too much noise or motion artifact. Transmissions documented heart rates ranging from 61 bpm to 276 bpm. Sinus rhythm was present in 102/238 (43%) of the transmitted tracings, followed by sinus tachycardia 69/238 (29%), SVT 39/238 (16%), and AF 19/238 (8%). Table 2 details the transmissions interpretations by patient diagnoses. SVT was captured in 3/10 SVT patients who transmitted tracings while AF was captured in 3/3 AF patients who transmitted tracings. No EAT, AT, or VT was captured in patients with those diagnoses who transmitted tracings. Additionally, initiation and termination of SVT were recorded in 10/238 (4%) transmitted tracings.

**Table 2:** Transmission interpretations according to patient diagnoses.

Patient Diagnoses (number of patients/number of transmissions)	Transmissions Interpretations (%)
Supraventricular tachycardia (10/157)	SVT 39 (25%)
	Sinus rhythm 62 (39%)
	Sinus tachycardia 49 (31%)
	Noise 7 (5%)
Atrial fibrillation (3/32)	AF 19 (60%)
	Sinus rhythm 10 (31%)
	Sinus tachycardia 3 (9%)
Ectopic atrial tachycardia (2/11)	EAT 0 (0%)
	Sinus rhythm 8 (73%)
	Sinus tachycardia 3 (27%)
Atrial tachycardia (1/3)	AT 0 (0%)
	Sinus rhythm 3 (100%)
Ventricular tachycardia (4/35)	VT 0 (0%)
	Sinus rhythm 19 (54%)
	Sinus tachycardia 14 (40%)
	Noise 2 (6%)

SVT, supraventricular tachycardia; AF, atrial fibrillation; EAT, ectopic atrial tachycardia; AT, atrial tachycardia; VT, ventricular tachycardia.

**Fig 1:**
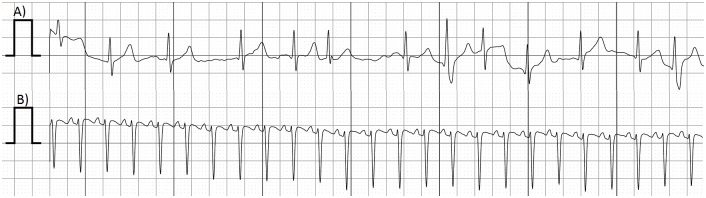
Samples of transmitted ECG tracings. A) Atrial fibrillation. B) Supraventricular tachycardia.

Detection of arrhythmias that prompted outpatient interventions occurred in 3/35 patients. Patient #1, an 18-year-old male with paroxysmal AF, had breakthrough AF episodes documented on transmitted tracings. The breakthrough episodes were successfully managed with “pill in the pocket” strategy. Patient #2, a 10-week-old female with neonatal SVT, had breakthrough SVT episodes while on propranolol. Her antiarrhythmic medications were up—titrated entirely as an outpatient resulting in control of her SVT. Finally, patient #3, a 1-year-old female with neonatal SVT, who had been managed with flecainide, had recurrence of tachycardia upon discontinuation of this medicine as documented by the transmitted tracings. We used the daily-transmitted tracings to monitor her QT interval during re-initiation of antiarrhythmic medications as an outpatient. The validity of using a one lead ECG as compared to a standard 12 lead ECG to monitor QT interval has been previously reported in the literature [4]. Fig 1 shows actual PDF printouts of the transmitted tracings.

### Survey Data

**Fig 2:**
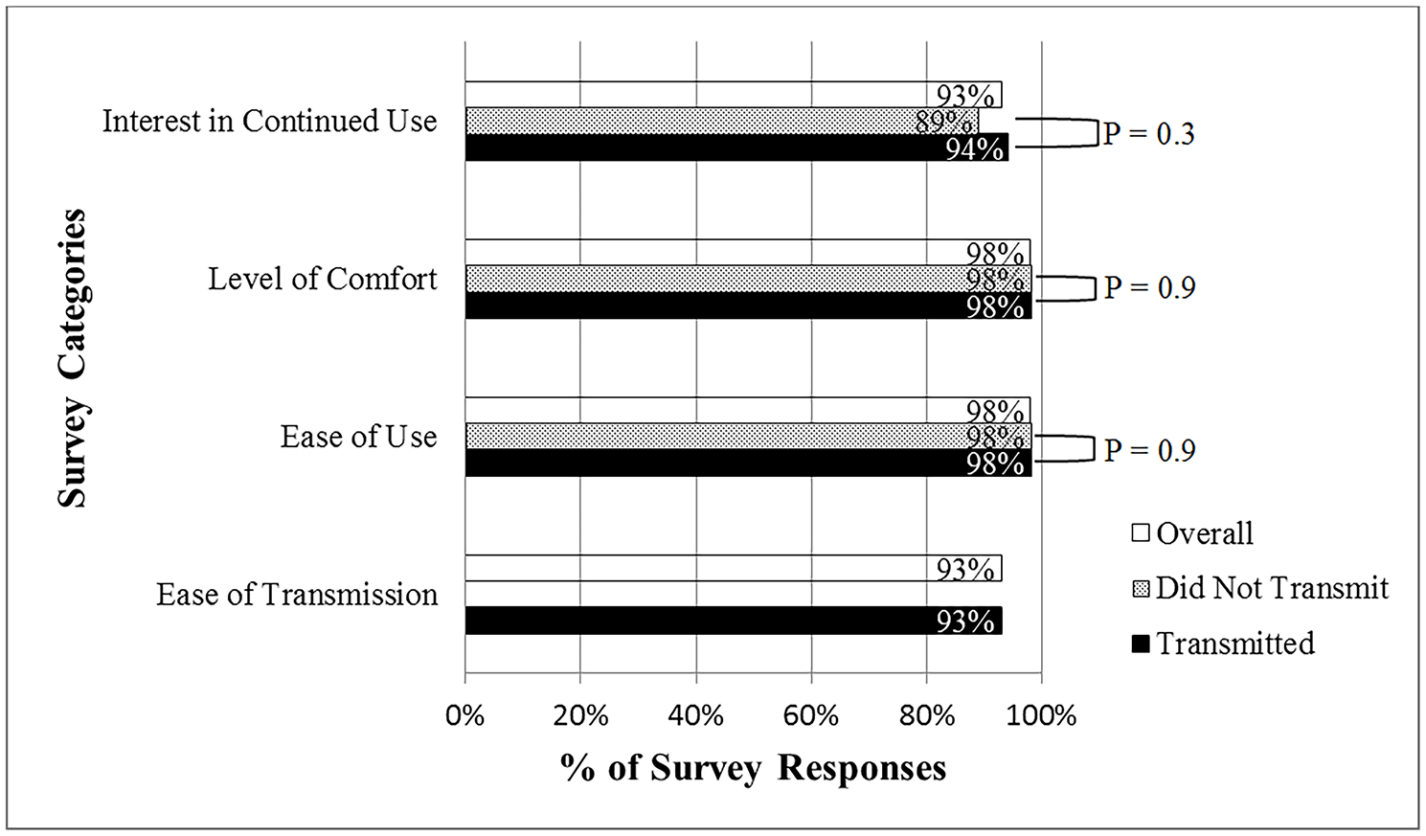
User satisfaction survey results. Comparison of different user satisfaction categories between those who transmitted and those who did not transmit.

A total of 18 parents and 10 patients (28/35 users) completed 126 surveys (1–12 surveys/user). Hand placement was used 78% of the times while chest placement was used in the remaining 22%. The median range of number of monthly-recorded tracings was between 0–4. Frequency of transmissions fluctuated not only from user to user but also over time. Of the 15 users who transmitted tracings and completed surveys, 5/15 (33%) users said they always transmitted recorded tracings, 3/15 (20%) users said they transmitted most of the recorded tracings, 2/15 (14%) users said they transmitted about half of the recorded tracings, and 5/15 (33%) users said they rarely transmitted the recorded tracings. 240 tracings were transmitted out of the 1700 estimated tracings that could have been recorded based on the median number of reported monthly-recorded tracings. User satisfaction with the device remained high over time (Fig 2). 98% of the survey responses indicated that it was easy to obtain tracings, 93% found it easy to transmit the tracings (from users who transmitted), 98% showed added comfort in managing their own or their child’s arrhythmia by having the device, and 93% showed interest in continued use of the device after the study period ended. Survey responses from users who transmitted tracings and users who did not transmit produced similar satisfaction scores. 98% vs 98% (P = 0.9) of the responses indicated that it was easy to obtain tracings, 98% vs 98% (P = 0.9) were more comfortable in managing arrhythmia, and 94% vs 89% (P = 0.3) were positive in continuing using the device.

In addition to completion of the survey, users also provided comments highlighting their experience with the device. While some users found it difficult to initiate tracings on an active infant or toddler, one mother was successful in obtaining tracings of diagnostic quality from her infant by swaddling the infant and holding the infant’s hands on the device. While some users found the need to alert the research team of their transmitted tracings cumbersome particularly after business hours, others cited that the device gave them greater peace of mind with the ability to promptly record the rate and rhythm leading to a prompt diagnosis thus avoiding them a trip to the emergency department.

## Discussion

This study is the first to investigate the usefulness of a smartphone-enabled ECG technology in the pediatric arrhythmia population. The data demonstrate that a smartphone-enabled ECG device can generate tracings of diagnostic and therapeutic quality in children with diagnoses of AF and SVT. In contrast to prior cautions against using previously developed smartphone-enabled heart rate detection technology to detect tachycardia in children with heart rate greater than 200 bpm [5], the AliveCor device consistently and accurately detected heart rate and rhythm in the mid to upper 200 bpm range, thereby enabling a practitioner to manage and treat pediatric arrhythmias remotely in real time. Potential uses for this device include school nurses offices where children without cellphones could transmit a tracing remotely.

The digital revolution, partly fueled by the development and widespread adoption of smartphones, has created enormous potential in the democratization of medical knowledge and technology with 90% of American adults owning a cell phone and 58% having a smartphone [6]. The pediatric population could have even greater benefit from smartphone-enabled technologies, as more teens and their caregivers have smartphones than the national average [7]. Data suggest that 85% of teens and caregivers use smartphones [8] and 95% of teens are now “online” [7]. Furthermore there is evidence that cardiac wireless remote monitoring with automatic clinician alerts reduces the time to a clinical decision in response to clinical events [9]. In this setting, the low startup cost, simplicity, and availability of this smartphone-enabled ECG technology make it ideal to leverage unprecedented large-scale data collection and analysis hopefully leading to high quality individualized care a decreased cost. This study demonstrates that smartphone-enabled ECG tracings achieved extremely high user satisfaction mainly by facilitating intensive outpatient monitoring and management of patients with arrhythmias. Still the question remains whether the widespread use of this technology will cause extra burdens to the medical system overall due to generated costs of its own, such as the need for an automated or on demand physician based ECG interpretation service.

In this study, the median range of number of monthly-recorded tracings remained low at 0–4. Additionally, 43% of users did not transmit any tracings throughout the entire study period while half of those who transmitted tracings indicated that they only do so about half of the times. In addition, only 240 tracings were transmitted out of the estimated 1700 tracings that could have been transmitted (estimation using the reported median number of transmissions). Furthermore, there was no statistically significant difference in user satisfaction scores between users who transmitted tracings as compared to those who submitted many ECGs. These data suggest that users who did not transmit any tracings during the entire study period were just as comfortable as users who did transmit in managing arrhythmias with the added help of the device. They were also as likely to continue using the device long term. These findings seem to indicate that concerns for frivolous use of the device did not actualize in this study. Possible explanations for these findings include the acquired knowledge about one’s own condition, the ability to objectively record patient’s heart rate or rhythm, as well as the reassurance that at any time, a cardiologist’s interpretation of ECG tracings could be obtained. These data indicate that when applied to a specific at risk population, smartphone–enabled ECG technology may be more cost effective compared to current methods of monitoring. Further cost effective analysis studies are needed.

### Study Limitations

This study has several limitations. First, there is a selection bias in targeting pediatric arrhythmia patients who already had access to smartphones. The exclusion of non-English speaking patients could also cause sampling bias and limit the generalization of our data. Non-English patients were excluded to limit language barrier as a confounder as the instructions of use of the device and surveys are in English. The ECG data, albeit diagnostic, was collected from a small number of patients (20) with known arrhythmia diagnosis. Future studies with increased power would be needed to apply our study’s findings to the population at large. Second, data analysis and interpretation were mainly derived from descriptive statistics that in turn depended on our de novo created questionnaire. One obvious limitation of the questionnaire was the inability to clearly tease out the confounding factors that could affect the frequency of transmissions. Third, despite the fact that 80% of users returned at least one survey, there were concerns of skewed survey data due to the lack of consistent periodic responses resulting in an inconsistent number of returned surveys per users. However, user satisfaction was tracked over the study period. Therefore a consistent positive user satisfaction reflected in the surveys over time was indicative of a true overall experience. A validated standardized questionnaire and perhaps incentivized responses would normalize data collection, facilitate and strengthen data analysis. Fourth, the logistics of the study did not permit 100% real time evaluation of the transmitted tracings. Users indicated that having to notify the research team of their transmitted tracings was inconvenient especially after business hours. However, this situation is not unexpected since the user would go through the usual steps to contact the physician on call just as any other patient or parent would after business hours. Overall, the study’s system remains more expedient than the traditional mechanisms as it allows users to give clinical data directly to the physician. In one mechanism patients/parents would have had to alert the provider on call and subsequently be directed to the local emergency department if deemed necessary for evaluation. Alternatively they would transmit a tracing from an event recorder to a central processing facility to be analyzed by a technician who would subsequently alert the physician on call should the tracing be deemed pathologic. The physician on call would then review the tracing to confirm the diagnosis. In both instances, there are considerable added lag times between the presentation and evaluation of patients. In any case, users were instructed not to wait for a response should they believe that they needed emergent help. Fifth, there was no VT/VF, AT or EAT captured during the study period restricting the study’s ability to comment on the device’s diagnostic ability of these potentially malignant arrhythmias. The diagnosis of sinus tachycardia in these patients was made after comparing the transmitted tracings with the patients’ previous 12 lead ECGs at baseline and during tachycardia. The final limitation of this study was that the ECG reader was not blinded to the patient’s diagnosis. However, we performed intra-reader variability without statistical significance between the first and repeated interpretations.

## Conclusions

The AliveCor device recorded pediatric patients’ heart rate and rhythm in a fast and reliable manner. It also provided both the users and physicians with convenient and almost real-time access to the clinical data. As such it has been useful in helping pediatric cardiologists manage the pediatric arrhythmia patients on an outpatient basis. The potential powerful applications along with the positive user satisfaction are encouraging in predicting the successful integration of this technology in clinical practice. As the medical infrastructure grows to accommodate this newer technology, future studies should examine how the technology impact healthcare cost by preventing unnecessary Emergency Department and physician’s office visits.

## Supporting Information

S1 File Transmitted ECGs Samples. Representative ECGs of each of the transmission interpretation recorded.
(PDF)

## References

[1] Moller JH, Allen HD, Clark EB, Dajani AS, Golden A, Hayman LL, et al Report of the task force on children and youth. American Heart Association. Circulation 1993; 88:2479–2486. 8222143 10.1161/01.cir.88.5.2479

[2] Zimetbaum P, Goldman A. Ambulatory arrhythmia monitoring: choosing the right device. Circulation 2010;12:1629–1636. 10.1161/CIRCULATIONAHA.109.925610 20956237

[3] Lau JK, Lowres N, Neubeck L, Brieger DB, Sy RW, Galloway CD, et al iPhone ECG application for community screening to detect silent atrial fibrillation: a novel technology to prevent stroke. Int J Cardiol 2013; 165:193–194. 10.1016/j.ijcard.2013.01.220 23465249

[4] Rimmer LK, Rimmer JD. Comparison of two methods of measuring the QT interval. Am J Crit Care 1998; 7:346–54. 9740884

[5] Wackel P, Beerman L, West L, Arora G. Tachycardia detection using smartphone applications in pediatric patients. J Pediatr 2014; 164:1133–1135. 10.1016/j.jpeds.2014.01.047 24655535

[6] Pew Research Internet Project. Mobile technology fact sheet. http://www.pewinternet.org/fact-sheets/mobile-technology-fact-sheet/. Accessed November 3, 2014.

[7] Singh A, Wilkinson S, Braganza S. Smartphones and pediatric apps to mobilize the medical home. J Pediatr 2014; 165: 606–610. 10.1016/j.jpeds.2014.05.037 24986454

[8] Pew Research Internet and American Life Project. Teens and Technology 2013. http://www.pewinternet.org/2013/03/13/teens-and-technology-2013/. Accessed November 3, 2014.

[9] Crossley GH, Boyle A, Vitense H, Chang Y, Mead RH, CONNECT Investigators. The CONNECT (Clinical Evaluation of Remote Notification to Reduce Time to Clinical Decision) trial: the value of wireless remote monitoring with automatic clinician alerts. J Am Coll Cardiol 2011; 57:1181–9 10.1016/j.jacc.2010.12.012 21255955

