# Supplementary material for: SPEAR Trial: Smartphone Pediatric ElectrocARdiogram Trial
Source: PLoS One. 2015 Aug 21;10(8):e0136256. doi: 10.1371/journal.pone.0136256 (PMC4546652; doi:10.1371/journal.pone.0136256)
Supplement: S1 File — Representative ECGs of each of the transmission interpretation recorded. (PDF) [file pone.0136256.s001.pdf]

Patient:
ID:
Recorded:
Heart Rate: 117 bpm **Duration:** 1mins

Symptoms: Palpitations, Dizziness, Shortness of breath, Fatigue

AliveCor™

Enhanced Filter, Mains filter: 60Hz Scale: 25mm/s, 10mm/mV

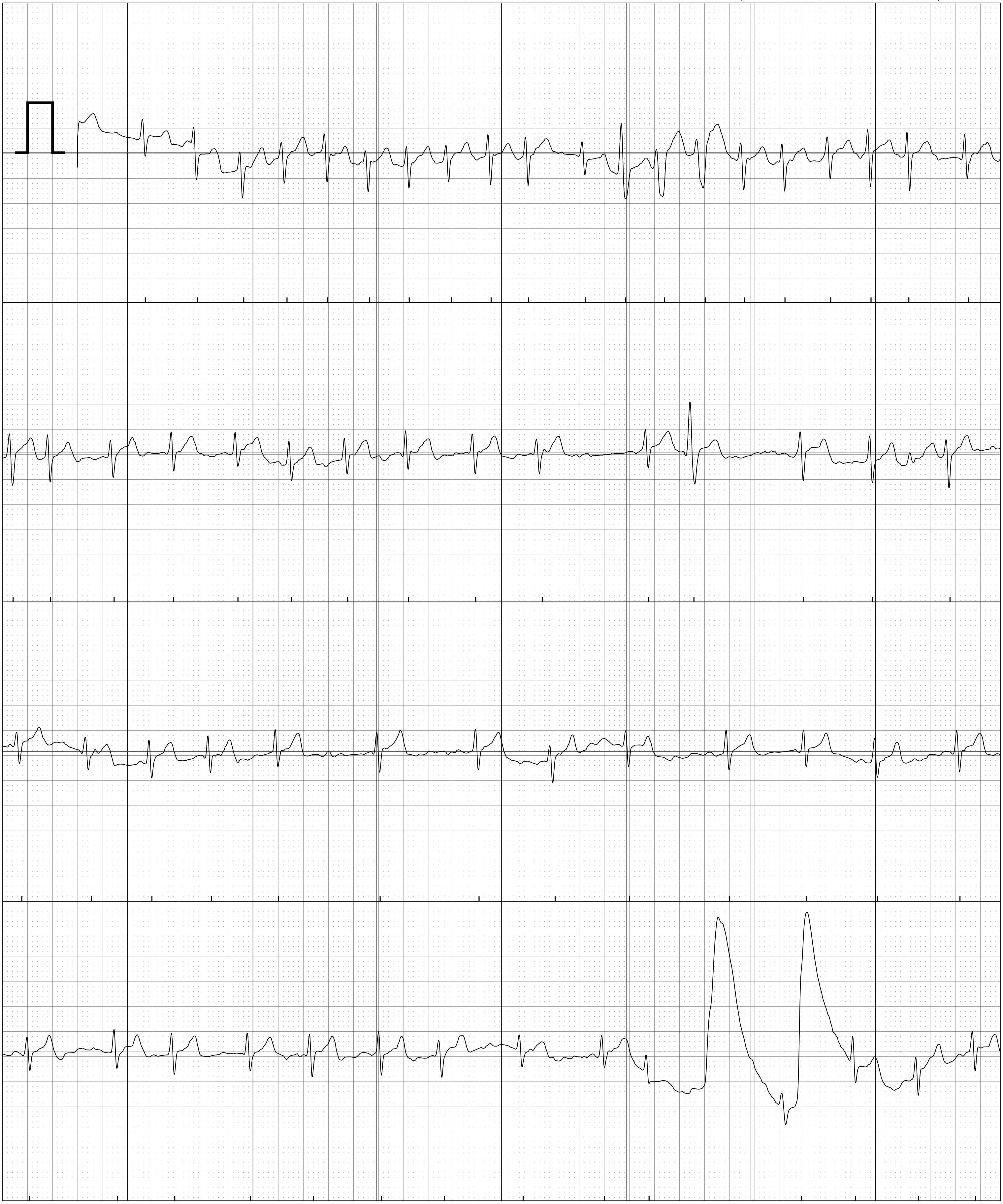

(c) Copyright 2012-2013, AliveCor Inc, AliveECG v1.5.0.1, Report v2.2.0, UUID: 81609F57-50B7-4AF0-AF25-1AB14962312F

Patient:
ID:
Recorded:
Heart Rate: 117 bpm **Duration:** 1mins

Symptoms: Palpitations, Dizziness, Shortness of breath, Fatigue

Enhanced Filter, Mains filter: 60Hz Scale: 25mm/s, 10mm/mV

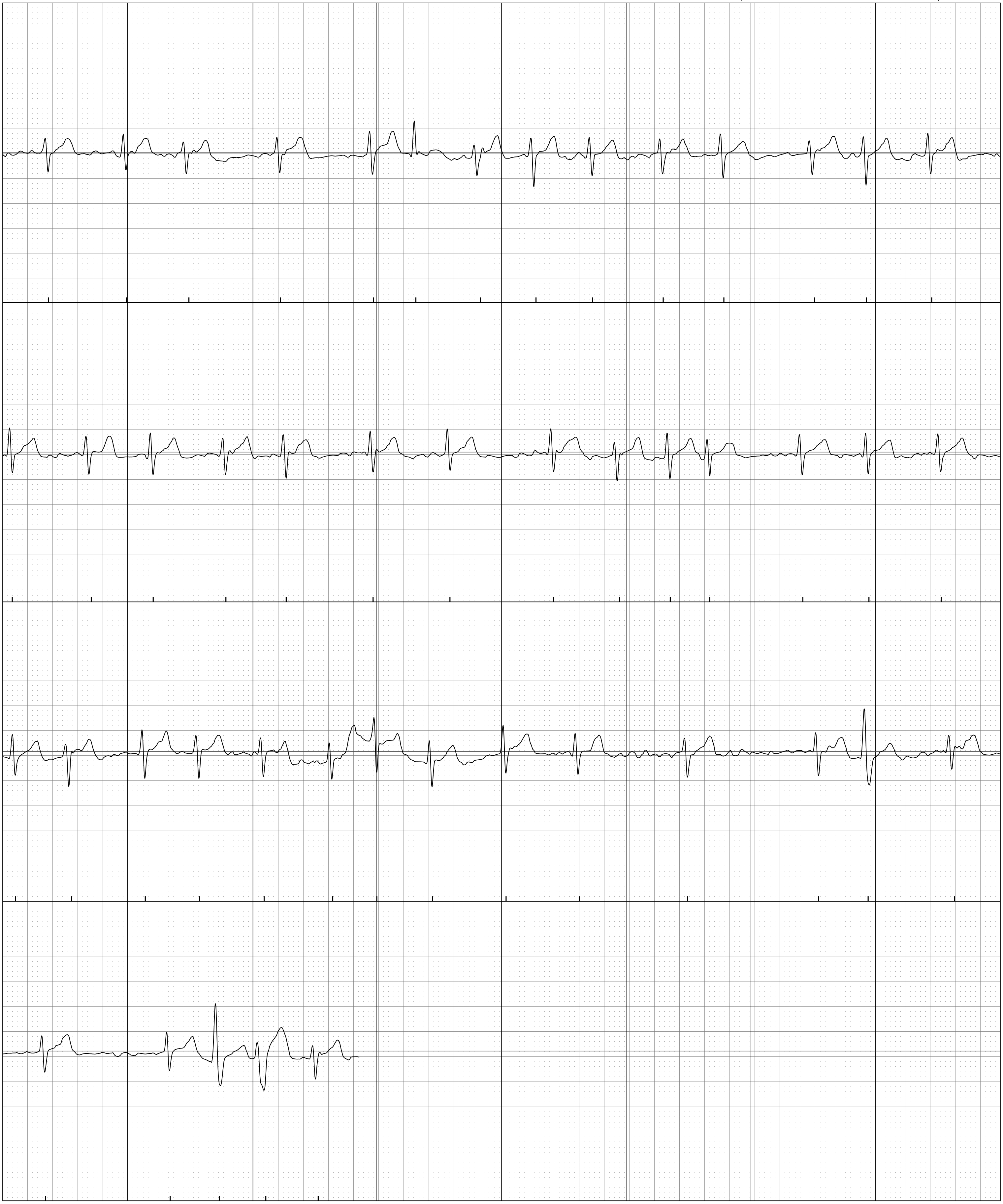

(c) Copyright 2012-2013, AliveCor Inc, AliveECG v1.5.0.1, Report v2.2.0, UUID: 81609F57-50B7-4AF0-AF25-1AB14962312F

Patient:
Recorded:
Heart Rate: 132 bpm **Duration:** 30s

AliveCor™

Mains filter: 60Hz Scale: 25mm/s, 10mm/mV

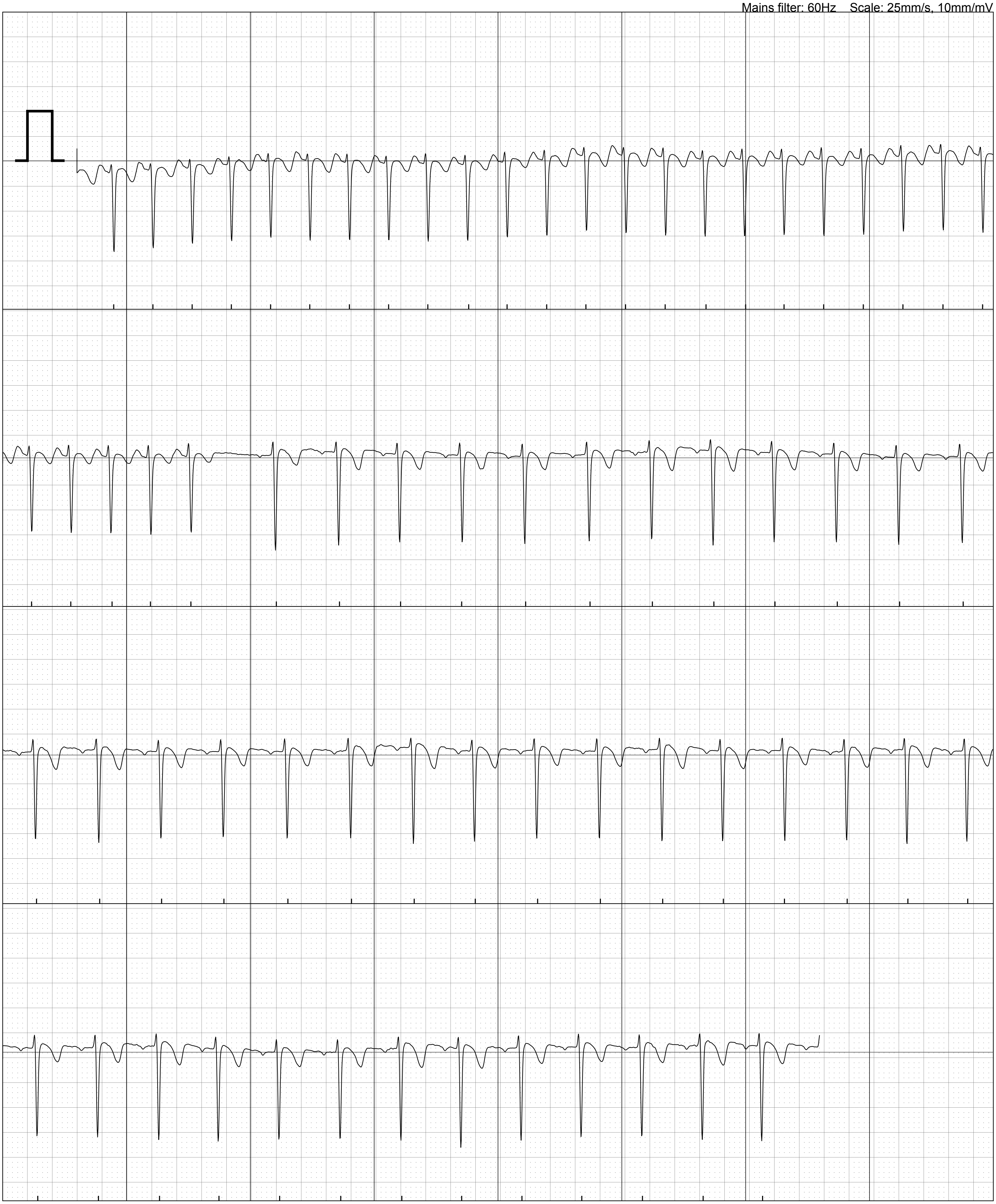

(c) Copyright 2012-2013, AliveCor Inc, AliveECG v2.0.0.1, Report v2.2.0, UUID: C2A003FD-96F3-4659-BEC3-A0A051C03F29

Recorded:
Heart Rate: 72 bpm **Duration:** 30s

AliveCor

Enhanced Filter, Mains filter: 60Hz   Scale: 25mm/s, 10mm/mV

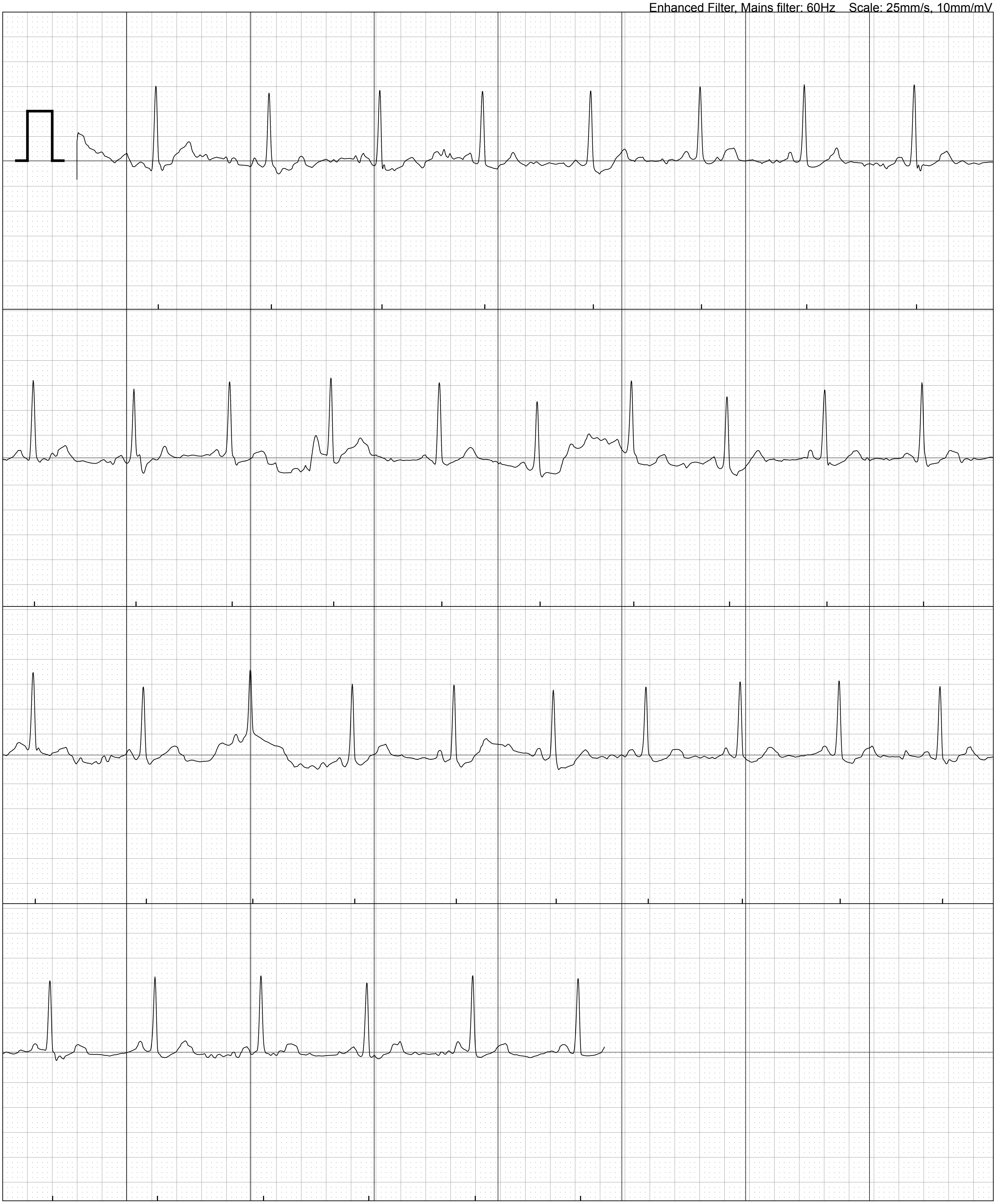

(c) Copyright 2012-2013, AliveCor Inc, AliveECG v1.5.5.0, Report v2.2.0, UUID: 73680EE5-DB3E-46DA-8122-BA35A6873F09

Patient:
Recorded:
Heart Rate: 276 bpm **Duration:** 30s

Symptoms: No symptoms

AliveCor™

Enhanced Filter, Mains filter: 60Hz Scale: 25mm/s, 10mm/mV

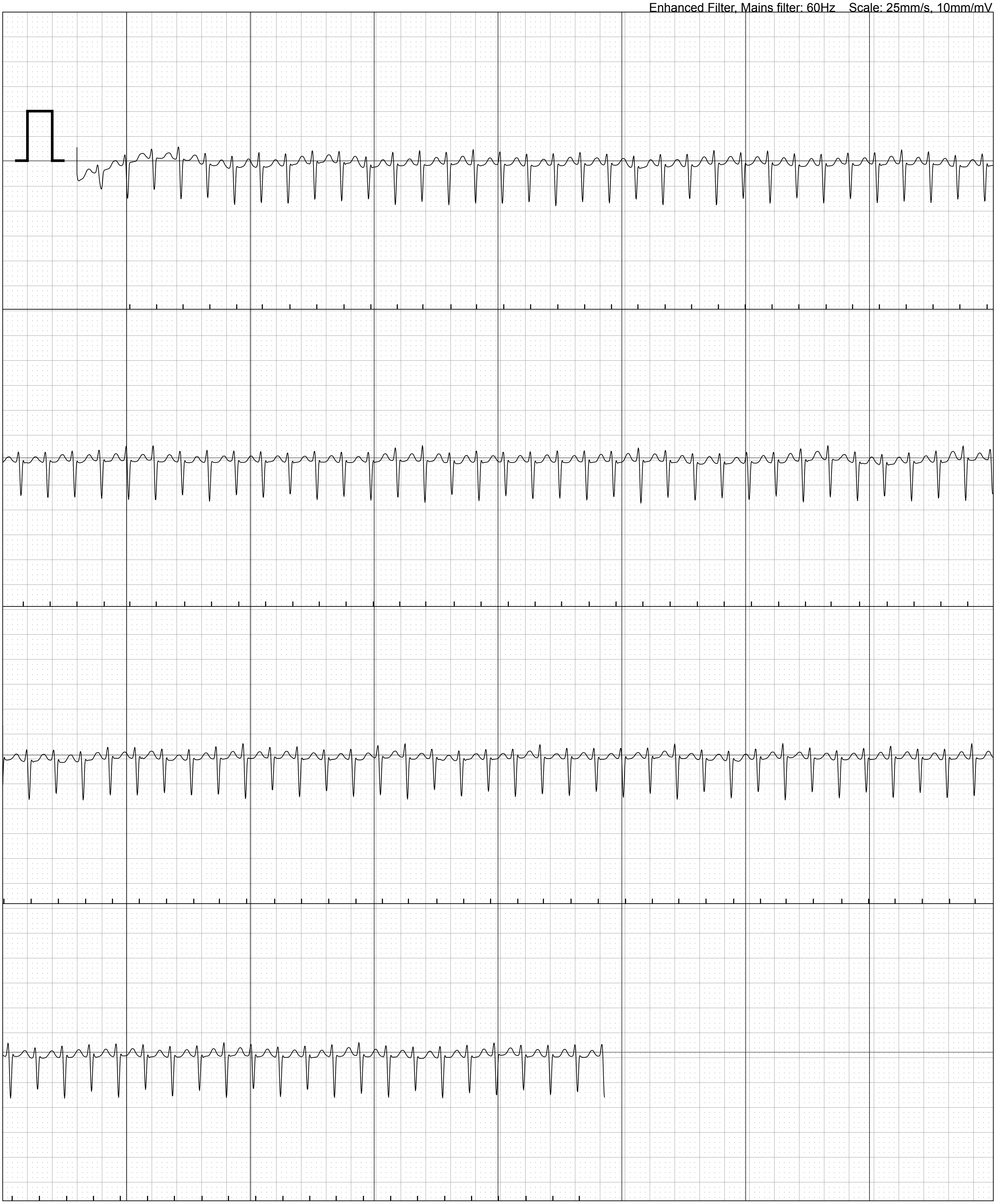

(c) Copyright 2012-2013, AliveCor Inc, AliveECG v2.0.1.1, Report v2.2.0, UUID: FA82E80E-7BF3-4EDE-A3C7-7DBDC29C300B

Patient:
Recorded:
Heart Rate: 112 bpm **Duration:** 1mins

Symptoms: No symptoms

AliveCor™

Enhanced Filter, Mains filter: 60Hz Scale: 25mm/s, 10mm/mV

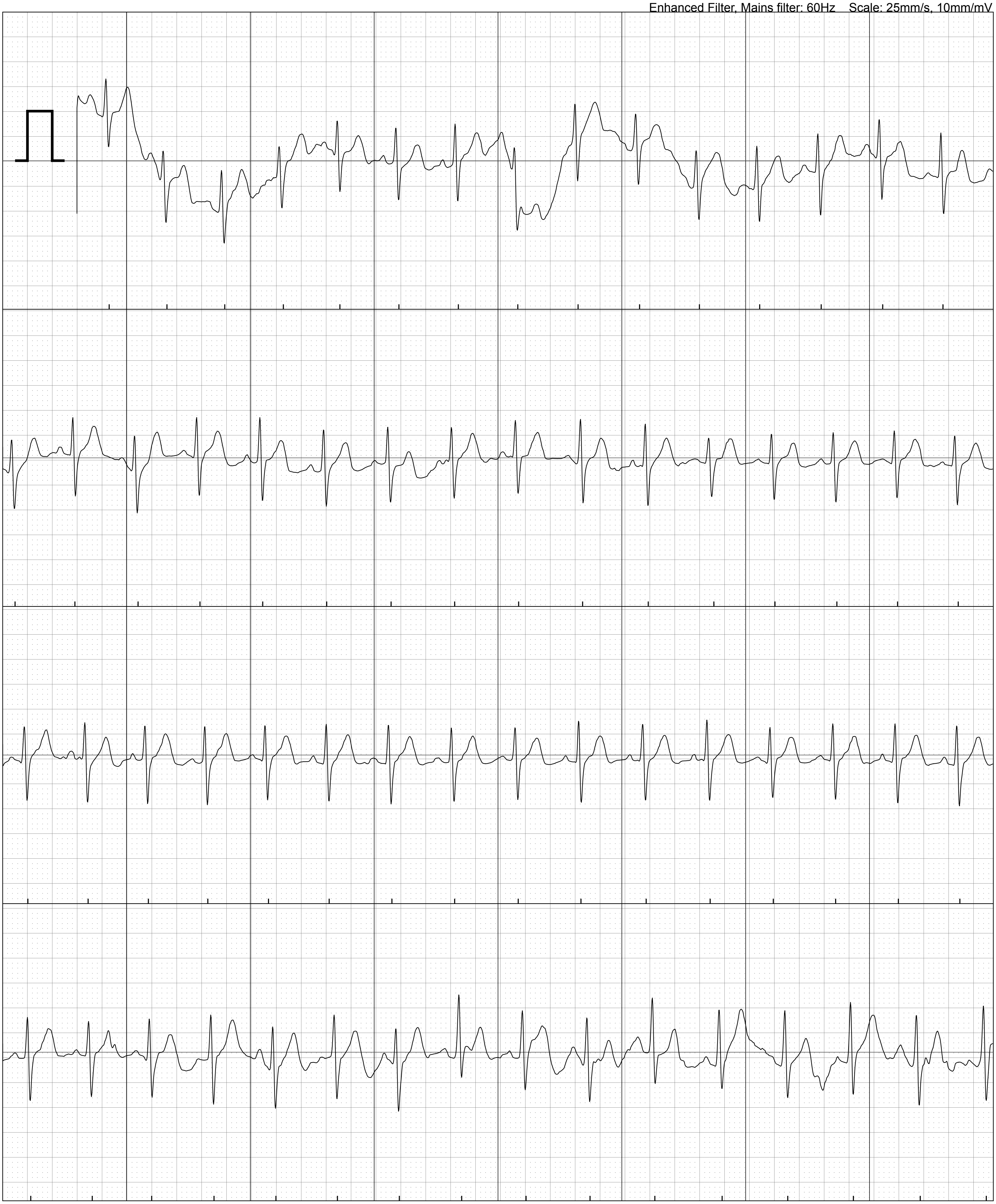

(c) Copyright 2012-2013, AliveCor Inc, AliveECG v1.5.0.1, Report v2.2.0, UUID: 7F47A1B9-E323-4B3D-840E-08125B518A5A

Patient:
Recorded:
Heart Rate: 112 bpm **Duration:** 1mins

Symptoms: No symptoms

AliveCor

Enhanced Filter, Mains filter: 60Hz Scale: 25mm/s, 10mm/mV

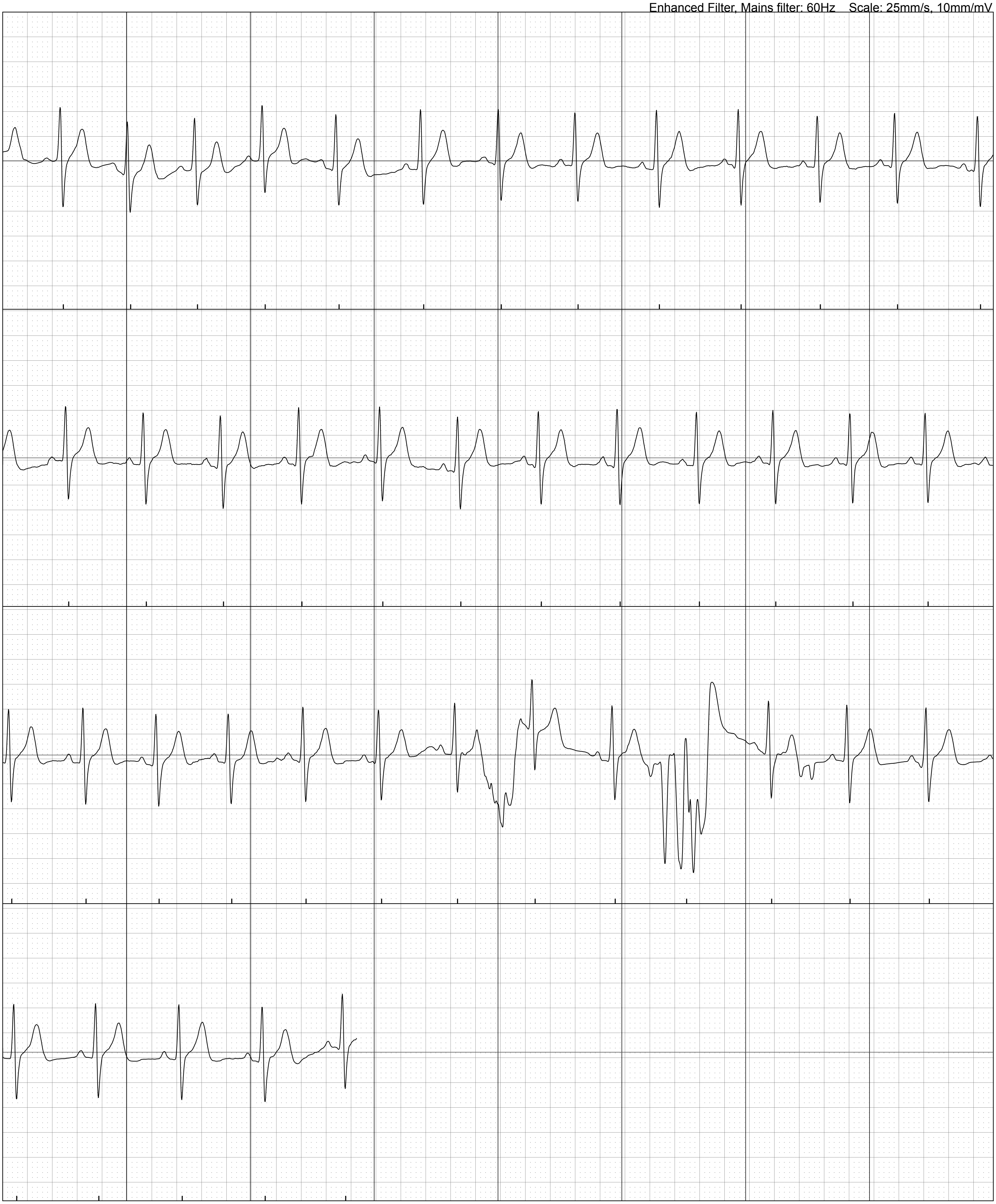

(c) Copyright 2012-2013, AliveCor Inc, AliveECG v1.5.0.1, Report v2.2.0, UUID: 7F47A1B9-E323-4B3D-840E-08125B518A5A
